# Static compression regulates OPG expression in periodontal ligament cells via the CAMK II pathway

**DOI:** 10.1590/1678-775720150156

**Published:** 2015

**Authors:** YI Jianru, LI MeiLe, Yan YANG, Wei ZHENG, LI Yu, Zhihe ZHAO

**Affiliations:** 1 - Sichuan University, West China School and Hospital of Stomatology, State Key Laboratory of Oral Diseases, Department of Orthodontics, Chengdu, China.; 2 - Sichuan University, West China School and Hospital of Stomatology, State Key Laboratory of Oral Diseases, Department of Oral and Maxillofacial Surgery, Chengdu, China.

**Keywords:** Osteogenesis, Periodontal ligament, Calcium-calmodulin-dependent Protein kinases, Mechanotransduction

## Abstract

**Objective:**

This study aimed to investigate the potential role of CAMK II pathway in the compression-regulated OPG expression in periodontal ligament cells (PDLCs).

**Material and Methods:**

The PDL tissue model was developed by 3-D culturing human PDLCs in a thin sheet of poly lactic-co-glycolic acid (PLGA) scaffolds, which was subjected to static compression of 25 g/cm^2^ for 3, 6 and 12 h, with or without treatment of KN-93. After that, the expression of OPG, RANKL and NFATC2 was investigated through real-time PCR and western blot analysis.

**Results:**

After static compression, the NFATC2 and RANKL expression was significantly up-regulated, while partially suppressed by KN-93 for 6 and 12 h respectively. The OPG expression was significantly down-regulated by compression in 3 h, started to elevate in 6 h, and significantly up-regulated in 12 h. The up-regulation after 12 h was significantly suppressed by KN-93.

**Conclusions:**

Long-term static compression increases OPG expression in PDLCs, at least partially, via the CAMK II pathway.

## INTRODUCTION

Mechanical loading has long been deciphered to regulate bone metabolism, and plays critical roles in orthodontic tooth movement (OTM)^4^ ^,^ ^10^ ^,^ ^22^. When teeth are subjected to orthodontic force, the remodeling of the adjacent periodontium is initiated. The static compression induces bone resorption at the pressure side, resulting in a “loose” tooth, followed by bone formation to complete a bone remodeling cycle, which restores the periodontium and strengthens the tooth again^29^. Lack of this recovery process may lead to alveolar bone loss followed by gingival recession and tooth removability.

The molecular mechanisms under bone remodeling in OTM has been extensively investigated. Numerous studies have focused on osteoclastogenesis, the rate-limiting step in OTM^1^ ^,^ ^12^ ^,^ ^16^. Particularly, COX-2 has been shown to induce up-regulation of receptor activator for nuclear factor-κ B ligand (RANKL), an essential pro-osteoclastogenic factor, in periodontal ligament cells (PDLCs) under compression^19^. However, little is known about the pathways to regulate the mechano-induced expression of osteoprotegerin (OPG), the decoy receptor of RANKL, which inhibits bone resorption while promoting bone formation^6^.

The Ca^2+^/calmodulin-dependent kinase (CAMK) family has been recognized as a key mediator in living organisms and various biological processes^9^. Recent studies have revealed its critical role in bone development and homeostasis. The CAMK II pathway has been found to regulate the RANKL-induced osteoclast formation via the cAMP-response element binding protein (CREB) pathway^3^. The involvement of CAMK II in regulating the RANK-MEK-ERK pathway has been detected^21^. Moreover, the CAMK II-CREB pathway was proposed to play an important role in bone homeostasis.

We previously established a periodontal ligament tissue model (PDLtm) to simulate the bioprocess at the pressure side of PDL^18^. Interestingly, through microarray screening, we found up-regulation of the CAMK II pathway in the loaded PDLCs^17^, which is known to respond to compression and result in OPG enhancement in osteoblast^11^. Therefore, in the present study, we aimed to investigate the potential role of CAMK II pathway in the expression of OPG and RANKL in the PDLtm under static compression.

## MATERIAL AND METHODS

### Preparation of PLGA scaffold

The poly lactic-co-glycolic (PLGA) polymers (10^5^ g/mol) were used for the synthesis of the scaffolds as previously described^17^. Briefly, 200 mg mixture of PLGA polymers and sucrose particulates with a volume ratio of 15:85 was applied with compression to form a thin sheet in a square mould (2 cm × 2 cm). Subsequently, the sheets were placed in a CO_2_ reaction kettle for 48 h, and the sucrose was then removed by immersing the PLGA sheets in ddH_2_O for 48 h. After that, the PLGA sheets (2 cm×2 cm×300 μm) were packaged and sterilized for experimental use.

### Establishment of the PDLtm

Five lines on human PDLCs were established following a well-documented method^17^. The periodontal tissue was collected from the teeth extracted for orthodontic reasons with the donors signing an informed consent form and the approval of the Institutional Review Board in our hospital. The PDLCs of the 3-6^th^ passage were used for this experiment. The 3-D PDLtm was established by dripping suspension of PDLCs into the PLGA sheet that was put in a 6-well plate, approximate 1×10^5^ cells in 2 ml medium *per* sheet. After 24 h, the PDLtm was displaced to another 6-well plate. Each cell line (n=5) was used for the experiment in three replicates, two of which were pooled together for western blot and one for real-time PCR.

### Histological observation

Four days after the establishment of PDLtm, the growth of PDLCs in scaffolds was investigated by microscopic observations. First, the PDLtm was stained by acridine orange (0.01%) and observed under a fluorescence inverted microscope (Leica DMI6000B, Germany). Second, the PLGA/PDLC construct was prepared for scanning of electron microscopy (SEM, Inspect F, FEI, USA)^17^.

### Application of compressive force

The PDLtms were randomly assigned into three groups, i.e., the control group, the compression group (Cg) and the compression+KN-93 (an inhibitor of the CAMK II pathway) group (CKg). To simulate the pressured periodontium in OTM, a modified “weight” method was used. Briefly, a cover glass and a bottle of granules were placed on the PDLtm to produce a compression of 25 g/cm^2^, which has been proved to be the optimal force level for this model^18^. In the compression+KN-93 group, 0.01 mM KN-93 (Sigma) was added to the media to suppress the CAMK II pathway.

### Real-time PCR

**Figure 1:**
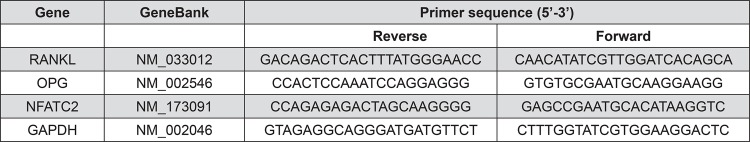
Primer used in real-time PCR analysis

Three, six and twelve hours after applying interventions, total RNA (n=5) was extracted by dissolving the PDLtm using TRIzol reagent (Invitrogen, Carlsbad, USA). The quality and integrity of extracted RNA samples were validated before their use. Real-time PCR was performed with a SYBR Green reaction Kit (Roche Diagnostics, China) in a LightCycler according to the manufacturer’s instruction to investigate the mRNA expression of RANKL, OPG, and Nuclear factor of activated T-cells (NFAT) C2. GAPDH served as the internal control. The sequences of relevant primers were shown in Figure 1.

### Western blotting

Twelve hours after force application, the total proteins (n=5) were collected using total protein extraction kit (Keygen Biotech, China). The 15 μL prepared samples (40 μg of protein) per lane were separated by SDS-PAGE and then transferred to the polyvinylidene difluoride (PVDF) membrane. After that, the PVDF membranes were probed with antibodies to RANKL (1:1000, Santa-Cruz, USA), OPG (1:1000, Santa-Cruz, USA) GAPDH (1:1000, Beyotime, China) overnight at 4°C. Subsequently, the membranes were immersed in secondary antibody (1:5000, Beyotime, China) for 1 h. The immunoreactive proteins were visualized by a chemiluminescence kit (Millipore). The band intensities were evaluated using Quantity One software (Bio-Rad, Hercules, USA).

### Statistical analysis

All data was expressed as mean±SD. The comparison among 3 groups was conducted by one-way analysis of variance (ANOVA) followed by LSD *post hoc* test using SPSS software of version 13.0. Differences with p<0.05 were set as significant.

## RESULTS

### The 3-D cultured PDLCs

**Figure 2:**
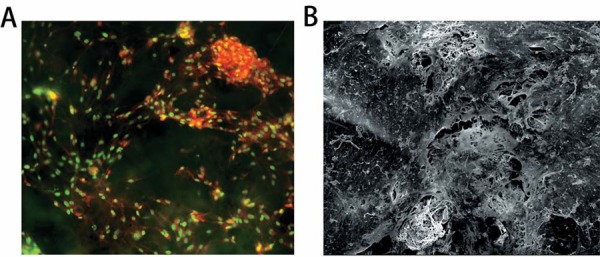
The microscopic observations of PDLtm. (A) Spindle PDLCs grew densely with nuclear in green-yellow and cytoplasma in orange-red, 200×; (B) The PDLCs, secreted extracellular matrix (SEM) and PLGA scaffolds were integrated, 300×

By acridine orange staining, the growth of PDLCs was observed under microscope (Figure 2A). In addition, the secreted extracellular matrix, the PDLCs and the scaffolds were observed to be interconnected under SEM (Figure 2B). More characterization of this PDLtm has been previously shown^18^.

### The NFATC2 expression

**Figure 3:**
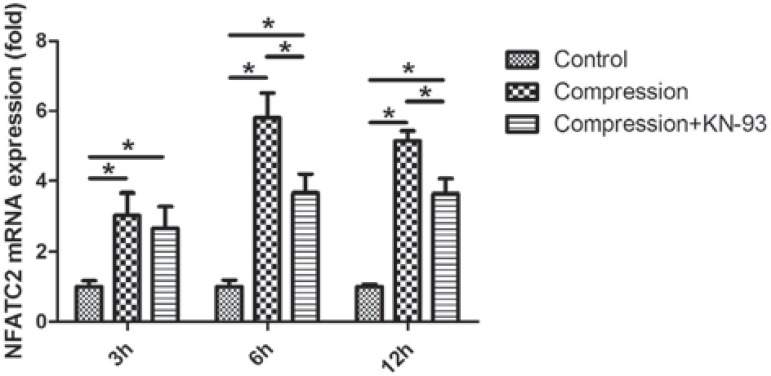
The time-course expression of NFATC2 in loaded PDLCs at mRNA level. Compression enhanced the NFATC2 expression throughout the experiment, while its expression was inhibited by KN-93 after 6 and 12 h. *p<0.05

Under static compression, the NFATC2 expression was significantly up-regulated, which peaked in 6 h (Figure 3). KN-93 partially suppressed the compression-induced up-regulation of NFATC2 in 6 and 12 h.

### The OPG/RANKL expression

**Figure 4:**
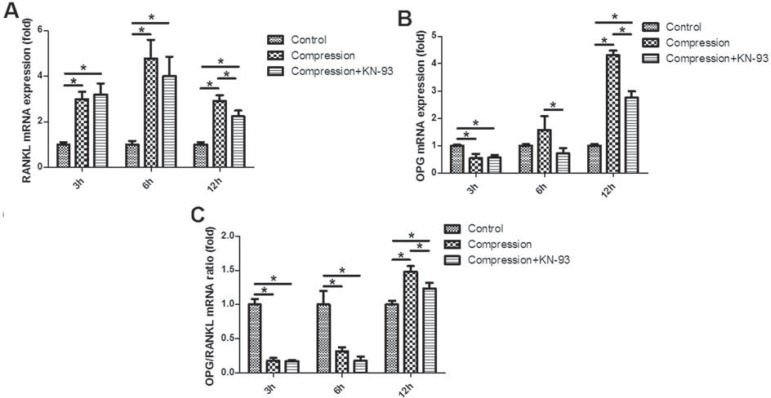
The time-course expression of osteoprotegerin (OPG) and RANKL in loaded PDLCs at mRNA level. (A) RANKL; (B) OPG; (C) OPG/RANKL ratio.*p<0.05

The real-time PCR analysis revealed the significant up-regulation of RANKL expression under static compression, which peaked in 6 h (Figure 4A). In contrast, the OPG expression was significantly down-regulated after 3 h, while up-regulated in 12 h (Figure 4B). The OPG/RANKL ratio was reduced in 3 and 6 h while enhanced in 12 h (Figure 4C).

Notably, after 12 h, the up-regulation of RANKL, OPG and OPG/RANKL ratio was partially suppressed by KN-93, suggesting that the CAMK II pathway took part in the up-regulation of RANKL and OPG in PDLCs induced by long-term stimulation of static compression (Figure 4).

**Figure 5:**
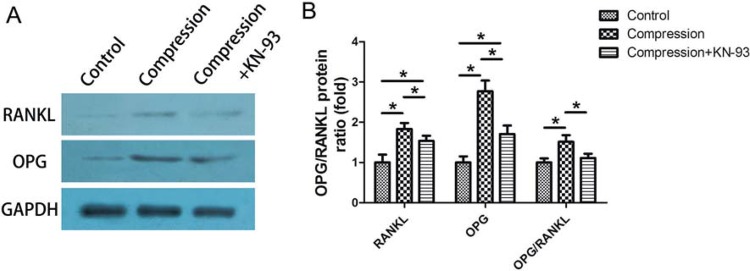
(A) The protein expression of osteoprotegerin (OPG) and RANKL in the loaded PDLCs after 12 h. The static compression significantly elevated the OPG and RANKL expression in PDLCs, while the enhancement was suppressed by KN-93; (B) The fold change of the expression of OPG, RANKL and OPG/RANKL ratio. *p<0.05

Moreover, the western blotting method showed results similar to the PCR assay, indicating that after 12 h of static compression there was an increased protein expression of RANKL and OPG, which was partially suppressed by KN-93 (Figure 5).

## DISCUSSION

Orthodontic tooth movement (OTM) has been generally featured with bone remodeling, a fundamental biological process consisting of bone resorption and bone formation^23^. Particularly, the initiated bone resorption at compression sites results in tooth movement, and the following bone formation surrounding roots strengthen the new position of the teeth^29^. Numerous studies focused on the osteoclastogenesis at the pressure side and sketched the contour of underlying mechanisms^12^ ^,^ ^13^, while the subsequent bone formation was less studied.

The CAMK family contains a series of proteins playing critical roles in bone modeling and remodeling. Afamin enhances osteoclastogenesis by decreasing intracellular cAMP levels via the CAMK pathways^14^. The role of the CAMK II pathway in the parathyroid hormone-related protein (PTHrP)-regulating osteoclast inhibitory lection has been recently identified^32^. Though KN-93 was observed to exert effects on other pathways^8^, it has been applied as a classic CAMK II pathway inhibitor in many studies^25^ ^,^ ^27^. Recently, by using KN-93, the enhancement of OPG in osteoblast under mechanical stimuli was suppressed^11^. However, limited information concerning its effect on OTM is available till now.

The microarray screening for gene expression profiles in our previous study has revealed the potential role of the CAMK II pathway in the mechanoresponse of PDLCs^17^. In the present study, PDLCs were embedded in 3-D PLGA scaffolds and cultured under static compressive force. The ratio of OPG/RANKL expression was significantly down-regulated 3 and 6 h after loading, indicating a potential role of osteoclastogenesis induction^24^ ^,^ ^31^. On the other hand, the OPG expression declined in 3 h, while started to elevate in 6 h and was significantly up-regulated after 12 h. As an important anti-osteoclastic and pro-osteogenic factor, the marked up-regulation of OPG after 12h indicates suppression for bone resorption^15^. However, this elevation was greatly impeded by KN-93, a specific CAMK II pathway inhibitor, suggesting that CAMK II pathway takes part in the OPG up-regulation induced by long-term static compression stimulation. Notably, the CAMK II pathway has also been reported to regulate the mechano-induced OPG enhancement in osteoblast^11^, which is to a great extent similar to PDL fibroblast^2^.

The RANKL–RANK–OPG axis mediates osteoclast formation through activation of RANK on the osteoclast precursors by RANKL^28^. Although the constitutional expression of OPG in PDLCs is much higher than RANKL^26^, numerous studies demonstrated the comparatively slight up-regulation of RANKL in PDLCs induced by compressive force could promote osteoclast formation and the following tooth movement^13^ ^,^ ^18^. Therefore, the large ratio of total OPG vs. RANKL seems meaningless accounting for osteoclastic induction. A reasonable explanation has been given that it could be due to the tight cell–cell contact between PDLCs and osteoclast precursors, which could create a favorable micro-environment for RANKL–RANK binding, thereby preventing the interaction of OPG with RANKL^5^.

On the other hand, at the sites away from the cell–cell contact area, the expression of OPG could play an important role. In contrast to the study reporting reduced or unchanged OPG expression in PDLCs under compression^20^, in the present study the OPG expression was significantly up-regulated after long-term mechanical stimuli, consistent with our previous data^18^. The delayed but marked increase of OPG could account for the subsequent bone formation at pressure periodontium, which prevents from alveolar bone loss and strengthens tooth again after movement. In this sense, targeting the CAMK II pathway might potentially benefit the OTM periodontium by stimulating OPG expression.

In the present study, we observed that the expression of NFATC2 in PDLCs was enhanced by compressive force while partially inhibited by KN-93 treatment (Figure 3). NFATC2 is a transcription factor which plays an undisputable role in the mechanoresponse of bone tissue^30^. As downstream factors of Wnt-Ca^2+^ pathways, both NFATC2 and CAMK II are involved in the Wnt-Ca^2+^ bone formation regulated by pathways^7^. Interestingly, Wnt-Ca^2+^ pathways were up-regulated when PDLCs were treated with compressive force in our previous study^17^. Therefore, the enhancement of OPG in PDLCs under compressive force might be regulated via Wnt-Ca^2+^ pathways, which should be further identified in future.

Last but not the least, it is interesting to compare the masticatory force and orthodontic force, both of which are transmitted to alveolar bone through PDL, while it results in opposite effects on alveolar bone metabolism. Obviously, the present theory of “compression-PDL-osteoclastogenesis” process cannot interpret this contradictory phenomenon. Based on our results, it is reasonable to speculate that the CAMK II pathways may be much more sensitive to masticatory force (intermittent force) than orthodontic force (static force), which should be further identified.

## CONCLUSION

The OPG expression was significantly up-regulated in PDLCs after long-term static compression stimulation, which is at least partially regulated by the CAMK II pathway. These results have enriched the present understanding to molecular mechanisms in bone remodeling modulation in OTM.
